# Compared efficacy and tolerance of the neuromuscular blockade induced by brand-name (Nimbex®) and generic (Cisatrex®) of cisatracurium in mechanically ventilated critically ill patients: a crossover double-blind randomized study

**DOI:** 10.11604/pamj.2020.37.346.24986

**Published:** 2020-12-15

**Authors:** Nesrine Fraj, Khaoula Meddeb, Abdelbaki Azouzi, Sana Romdhani, Helmi Ben Saad, Mohamed Boussarsar

**Affiliations:** 1 Medical Intensive Care Unit, Farhat Hached University Hospital, 4000, Sousse, Tunisia,; 2 Research Laboratory N° LR12SP09, Heart Failure Farhat Hached University Hospital, 4000, Sousse, Tunisia,; 3 Laboratory of Physiology and Functional Explorations, Farhat Hached University Hospital, 4000, Sousse, Tunisia

**Keywords:** Generic drugs, cisatracurium, neuromuscular blocking, hypoxemic acute respiratory, neuromuscular blocking agents, pharmacodynamics

## Abstract

**Introduction:**

use of generic drugs is common. However, there is still concern among patients and physicians that brand name drugs are more efficient. The aim of the study was to compare efficacy and tolerance between two forms of cisatracurium: brand name versus generic name.

**Methods:**

it´s a crossover, randomized, double-blind physiological trial. Patients admitted for hypoxemic acute respiratory failure with PaO2/FIO2 < 200mmHg despite optimized ventilation and sedation thus requiring non-depolarizing neuromuscular blocking agents (NMBAs), were enrolled. Patients received consecutively, in a random order, cisatracurium brand name (Nimbex®) and generic (Cisatrex®) over two-hour period separated by one-hour washout period. Neuromuscular function was monitored by a calibrated train-of-four (TOF) stimulation device. Paralysis time delay to reach TOF of 2/4, recovery kinetics and tolerance were monitored. The number needed to demonstrate a significant difference in time delays to reach a TOF of 2/4 between the two forms of cisatracurium was estimated at 22 patients.

**Results:**

twenty-two patients were included. Eight (36.4%) had acute respiratory distress syndrome; 8(36.4%), acute exacerbation of chronic obstructive pulmonary disease and 3(13.6%), status asthmaticus. Median [IQR] SAPS II at admission, 28.5 [22, 41]. PaO2/FIO2, 121 [81, 156] mmHg. Paralysis time delays were respectively, 80 [50, 112] vs. 87 [65, 115] minutes, in Nimbex® group and Cisatrex® group; (p=0.579). Within the recovery period, the between two-studied drugs´ difference in TOF was at 0.25±0.96; p=0.64. There were no significant hemodynamic differences.

**Conclusion:**

the present study revealed no significant differences in efficacy nor in tolerance between cisatracurium brand name Nimbex® and generic name Cisatrex® in hypoxemic ventilated patients.

## Introduction

Generic drugs are made to be chemically and therapeutically equivalent to the brand name ones. These equivalencies are obtained at lower costs making generic drugs use certainly appealing. Therefore, many payers/providers have encouraged substitution of brand name drugs with inexpensive bioequivalent generic versions [1]. In the last 30 years, several controversies concerning generic drugs legislation have arisen. Recent literature reviews demonstrated that physicians, pharmacists and general population hold negative perceptions and knowledge of generic medicines [2-5]. Neuromuscular Blocking Agents (NMBAs) are usually used in patients with altered respiratory mechanics properties such as Acute Respiratory Distress Syndrome (ARDS), status asthmaticus or severe acute exacerbation of COPD (AECOPD) [6, 7]. Only few products exhibit the so called ideal Neuromuscular Blockade (NMB) effect which is influenced by several factors namely, required speed of onset and offset, cardiovascular stability, possible accumulation of the NMBA metabolites, elimination routes, and costs [8, 9]. One of these products is cisatracurium which is a non-depolarizing NMBA, a benzylisoquinolinium like atracurium, doxacurium, and mivacurium [10].

Since its introduction in the early 2000, cisatracurium was widely used in Intensive Care Units (ICUs) in developed countries. However, it generated relatively high costs [11]. Here and there several cisatracurium generics have been developed to respond to the need of lowering expenses. Cisatracurium became of relatively frequent use in critical care, taking advantage of its higher potency and limited side effects [12]. Payen *et al*. [13], reported that NMBAs were used in 9% of patients on day two, 7% on day four and 5% on day six. Cisatracurium accounted for 70% of NMBA use. In developing as in developed countries, cisatracurium use remained rather sporadic [4]. Recently the introduction of Cisatrex®, the generic of the brand name Nimbex®, provided an opportunity to substitute commonly used NMBAs for cisatracurium. Because of the physicians´ reluctance to prescribe generics due to different controversies, authors hypothesized that a proof of safety and efficacy of Cisatrex® compared to its brand-name could reassure intensivists. The aim of the present study was to compare efficacy and tolerance of two marketed forms, brand-name (Nimbex®) and generic name (Cisatrex®) of cisatracurium-induced paralysis in hypoxemic ventilated patients.

## Methods

**Study design and setting:** this was a crossover randomized double-blind physiological study conducted in an 8-bed Medical ICU from February 2015 to March 2016, which compared neuromuscular blockade efficacy and tolerance induced respectively by continuous perfusion of brand-name (Nimbex®) and generic name (Cisatrex®) of cisatracurium during two successive time periods.

**Inclusion criteria:** patients admitted to the ICU for severe acute respiratory failure with severe hypoxemia (PaO2/FiO2 < 200mmHg), presenting important patient-ventilator asynchrony under invasive mechanical ventilation, despite deep analgo-sedation as assessed by Ramsay Sedation Scale (RSS) [14], were enrolled when drug induced paralysis was required. Patients with history of allergy to cisatracurium, malignant hyperthermia, pregnancy, neuromuscular disorders or hemodynamic instability, were not included in the present study.

**Ethics:** prior approval of the Local Medical Ethics´ and Research Committee of and written informed consent from family members or surrogates were obtained.

**Trial registration:** isrctn.com ISRCTN89942618. Registered 30^th^ October 2017.

**Sample size:** it was assessed as the number of patients needed to demonstrate a difference (d) of 10 min in the mean paralysis time delay to reach a defined objective of paralysis assessed by a Train-Of-Four (TOF) ≤2/4. Given, Âµ1= mean delay for Nimbex® to reach TOF=2/4, assessed at 70mn after a trial on a pre-protocol patient. Âµ2= mean delay for Cisatrex® set at 60mn as approximately reported in literature [15]. d=Âµ1-Âµ2=70-60=10mn. σ=10mn=Standard deviation. Thus, variance is s2=100mn2 (variable distribution was normal and variances were equal). β=0.1 (Thus power of the study is 90%). According to normal distribution: α=5%; Z (1-σ)=1.96; β=10%; Z (1-β)=1.28. The sample size of each group was estimated according to the following formula [16]: =[((1.96+1.28)2) (102+102)]/102=22 patients in each group. The total sample size (two groups) was estimated at 44 patients. Taking into account the crossover design of the present study the number needed to treat was assessed at 22 patients.

**Data collection:** all data regarding patients´ characteristics at ICU admission, demographic characteristics, underlying diseases, diagnosis at admission, severity of illness, therapeutic characteristics, were collected. Chart abstractors were well trained residents. Before enrollment; clinical, physiological, therapeutic and outcome characteristics were collected at baseline after a short period of respiratory and hemodynamic stabilization. An explicit protocol was used to precise all needed definitions and to ensure uniform handling of collected and measured data. A data form was designed for this purpose.

**Studied medications:** cisatracurium is a non-depolarizing agent that acts as a competitive antagonist of nicotinic receptors, blocking the action of acetylcholine [17]. It´s a benzylisoquinolinium used as a neuromuscular-blocking drug similarly to atracurium, doxacurium and mivacurium [10, 17]. Nimbex®, the brand name of cisatracurium 2mg/ml, is a trademark of the Glaxo group of companies, AbbVie Corporation licensed use. *Active substance:* cisatracurium besilate, 6.70mg/1ml or 5mg/1ml expressed in cisatracurium base. *Excipients:* besilic acid at 32% (qsp pH 3 a 3.8), water for injection. Cisatrex®, 10mg/5ml, producted by MédiS, the generic of cisatracurium. *Active substance:* cisatracurium besilate 13.4mg (10mg base). *Excipients:* benzene sulfonic acid 20, water for injection.

**TOF application device:** TOF monitor (Innervator NS252FBB, Fisher-Paykel Health Care, New Zealand) was applied to test twitches of the thumb muscles (number and amplitude) in response to a peripheral neuro-stimulator of the ulnar nerve with a stimulus intensity set at 60mA. Neuromuscular block depth induced by neuromuscular blockade drugs was considered adequate when a target of two responses on the TOF was reached in order to reduce patient-ventilator asynchrony [18, 19].

**Protocol description:** a pre-protocol test was performed on a patient presenting enrollment criteria in order to define the minimum paralysis time delay, recovery time, best intervals for monitoring while training the co-investigator resident (NF) to monitor the different parameters and get familiar with the data collection form. The same co-investigator monitored all included patients to ensure maximal consistency and homogeneity of collected data. After one hour of stabilization period and referring to current state-of-the-art, targeting respiratory and hemodynamic stability, patients were randomized following a double-blind inclusion method, for the two products order of administration, based on a random table. The principal investigator (MB) implemented a random allocation sequence, enrolled and assigned participants to interventions. Table the assigned drug and concealed the sequence until all interventions were performed. Co-investigator (NF), participants and care providers were blinded after assignment to interventions.

Minimal patient-ventilator interactions were ensured in all studied patients within the study period. Paralysis depth was monitored by TOF [20]. A short period of stabilization under effective analgo-sedation as assessed by RSS [14] was performed. Midazolam and Remifentanyl is the biotherapy, usually used in the ICU, especially in COPD patients. When targeted levels of sedation are not reached, sedation could be switched to propofol and/or fentanyl mainly in ARDS patients. The neuromuscular blockade drug was initiated, continuous infusion of cisatracurium was started at 0.06mg.kg-1.h-1 and increased in increments of 0.03mg.kg-1.h-1 every 30 minutes to reach and sustain a TOF at 2/4, with a maximum study time limited to two hours and a maximum dose of 0.18mg.kg-1.h-1 as assessed by the pre-protocol test patient and The 2002 “Clinical Practice Guidelines for Sustained Neuromuscular Blockade in the Adult Critically Ill Patient” updated in 2016 [19].

During this same period, the following parameters were measured at equal intervals every five minutes: TOF, heart rate, systolic and diastolic blood pressures, and ventilatory parameters. The infusion of the first product was stopped after two hours to allow elimination (of the first active paralysis agent) before infusion of the second drug (wash-out period). The one-hour wash-out period was chosen based on the pharmacodynamic properties of the product which suggest that its elimination would be rapid due to its metabolism by Hofmann elimination [21] and as implied by several studies which assessed a recovery time ranging from 45 min to 68 min [15, 22-24]. The wash-out period was checked to be quite sufficient to recover a TOF of 4/4 as demonstrated in the pre-protocol test patient. This same period also allowed to monitor the recovery kinetics (recovery time) of the first product. At the end of the wash-out period, the second product was then introduced. Its effect and tolerance (hemodynamic tolerance and drug interaction) were monitored according to the same protocol. Next to the TOF, the monitoring of the paralysis was also assessed by the monitoring of clinical parameters, ventilatory parameters and patient-ventilator asynchronies.

**Primary outcome:** paralysis time delay. It is the time needed from cisatracurium intravenous infusion onset to reach a TOF of 2/4. It was also assessed the respective differences at each time intervals from the cisatracurium intravenous infusion onset until 120 min.

**Secondary outcomes:** recovery time. The time needed to reach a TOF of 4/4 after stopping the cisatracurium intravenous infusion. For commodity reasons, authors rather assessed the respective differences at each time interval from the cisatracurium intravenous infusion cessation until 60 min, especially between the two studied drugs´ TOF difference at 60 min of the recovery period. TOF variability: It was defined by the changes in the TOF responses between the different interval times within the paralysis period and recovery time. This would gauge the stability of the paralysis or decurarization over time [9]. Tolerance: Hemodynamic tolerance was defined by a significant variation in heart rate above 30% from the baseline value and/or a significant drop of systolic and/or diastolic blood pressures above 30% from the baseline values. Drug interactions mainly with antibiotics was also used to evaluate tolerance.

**Definitions:** SAPS II (Simplified Acute Physiology Score): was used to measure disease severity for patients admitted to the intensive care unit [25]. Ramsay Sedation Scale (RSS). The RSS was the first scale to be defined to monitor the depth of sedation in the critically ill patient [14]. Performed using a series of steps: observation of behavior (score 1 or 2), followed (if necessary) by assessment of response to voice (score 3), followed (if necessary) by assessment of response to loud auditory stimulus or light glabellar tap (score 4 to 6).

**Statistical analysis:** variable distribution analysis was tested using the Kolmogrov-Smirnov test. Results were expressed as mean ± standard deviation (SD) (95%CI, confidence intervals) when the distribution was normal and variances were equal. If not, results were expressed by their medians (IQR, interquartile range). Qualitative data were expressed by their relative proportions. Time delays were compared between the two studied drugs and at each time intervals by applying repeated measures analysis of variance (ANOVA). Differences were tested meaning two-tailed “t” paired test. Statistical analyses were performed using the statistical software package SPSS20.0. The p values less than 0.05 were considered as statistically significant.

## Results

**Figure 1:**
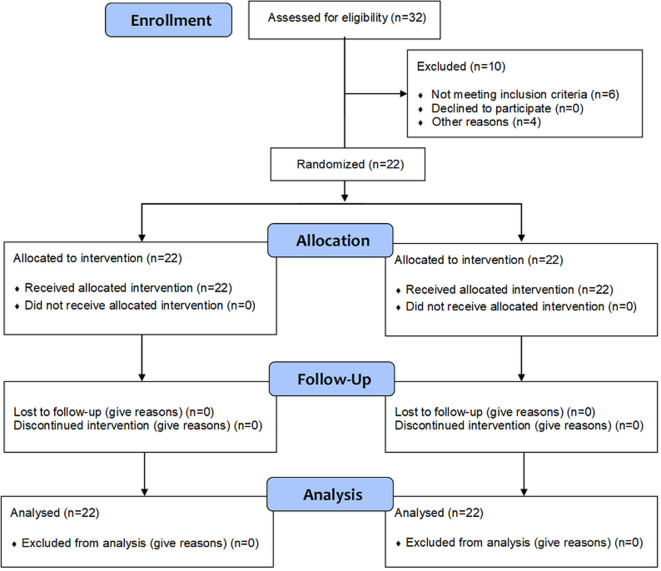
patients’ flow diagram

Patients´ flow is displayed on Figure 1. Within the study period, 32 patients were assessed for eligibility. Six were not included because of hemodynamic instability and four additional patients for organizational reasons. Patients´ characteristics are displayed in Table 1. Its main conclusions were: i) chronic obstructive pulmonary disease (COPD) was the most frequent underlying disease; ii) the main diagnosis at admission was balanced between ARDS and AE/COPD and iii) patients were quite severe as assessed by SAPS II at admission, length of stay and mortality; vi) several antibiotics have been prescribed with a potential risk of interaction with cisatracurium.

**Table 1:** patients’ demographic, clinical characteristics and outcome

Items	n=22
**Age** (years)a	55[40,62]
**Sex**, (Male)b	14(63.6)
**Weight** (kg)a	77.5[68,92]
**Underlying diseases**	
COPDb	8(40.9)
Asthmab	3(13.6)
Miscellaneousb	6(27.3)
Othersb	5(22.7)
**Diagnosis at admission**	
ARDSb	8(36.4)
AE/COPDb	8(36.4)
Status asthmaticusb	3(13.6)
Othersb	3(13.6)
**Illness severity at admission**	
SAPS IIa	28.5[22,41]
PaO2/FiO2a	121[81,156]
**Antibioticsb**	
Cefotaxime	7(31.81)
Imipenem-cilastatin	7(31.81)
Amikacin	3(13.63)
Teicoplanin	2(9.09)
Ciprofloxacine	2(9.09)
Gentamicin	2(9.09)
Colistin	2(9.09)
**Mechanical ventilation** (days)a	12[5,20]
**Length of stay** (days)a	14.5[6,20]
**Mortality**b	14(64%)

AE/COPD: acute exacerbation on chronic obstructive pulmonary disease; ARDS: acute respiratory distress syndrome; COPD: chronic obstructive pulmonary disease; ICU: intensive Care Unit; PaO2/FiO2: ratio of arterial oxygen partial pressure to fractional inspired oxygen; SAPS II: simplified acute physiology score ii. Median [Interquartile Range]; b, n(%).

Table 2 displays the patients´ clinical, therapeutic and ABG´s (arterial blood gases) characteristics at baseline. Indeed, the short period of stabilization, then the hour of the washout period, enable achieving near identical parameters which were mandatory to assure the comparability of the respective studied drugs. Furthermore, it was worthy of note that the patients displayed poor visco-elastic properties as demonstrated by the elevated Peak and Plateau pressures. PaO2/FiO2 was rather severe and the elevated PaCO2 may be explained by the important proportion of COPD patients and low Vt protective ventilation related permissive hypercapnia in ARDS patients. All patients experienced no significant modifications neither in clinical nor in ventilatory parameters. No patient-ventilator asynchronies have been detected in any patients within the protocol experimental period.

**Table 2:** patients’ clinical, therapeutic and ABG’s characteristics at baseline^a^

Items	Baseline 1 (n=22)	Baseline 2 (n=22)
**Ventilatory settings**		
**VT** (ml)	502[450,562]	490[450,562]
**RR** (c/min)	20[16,22]	20[16,22]
**PEEP** (mmHg)	4[0,8]	4[0,8]
**FiO2**	90[57,100]	100[50,100]
**Ventilatory parameters**		
**Ppeak** (cmH2O)	36[31,40]	36[31,41]
**Pplat** (cmH2O)	22[20,26]	24[22,30]
**AutoPEP** (cmH2O)	7[4,9]	6[4,9]
**ABG’s**		
**pH**	7.35[7.25,7,40]	-
**PaCO2** (mmHg)	53[39,68]	-
**PaO2/FiO2**	121[81,155]	-
**SBP** (mmHg)	127[114,148]	128[109,150]
**DBP** (mmHg)	70[60,84]	70[60,80]
**Vasopressive drugs** (µg/kg/min)	0.13[0,0.40]	0.13[0,0.46]
**Analgo-sedation**		
**Remifentanyl** (µg/kg/min)	0.13[0.13,0.19]	0.12[0.09,0.19]
**Midazolam** (µg/kg/min)	1.78[1.53,2.67]	1.78[1.53,2.67]
**Ramsay Sedation Scale**	5	5

Data are presented as Median [Interquartile range]. ABG’s: arterial blood gases; AutoPEEP: auto-positive end expiratory pressure; baseline 1: before the first drug; baseline 2 :before the second drug; DBP: diastolic blood pressure; FiO2: fraction of inspired oxygen; PaCO2: arterial partial pressure of carbon dioxide; PaO2: arterial partial pressure of oxygen; PaO2/FiO2: ratio of arterial oxygen partial pressure to fractional inspired oxygen; PEEP: positive end expiratory pressure; pH: potential of hydrogen; Ppeak: peak inspiratory pressure; Pplat: plateau pressure; RR: respiratory rate; SBP: systolic blood pressure; VT: tidal volume.

**Figure 2:**
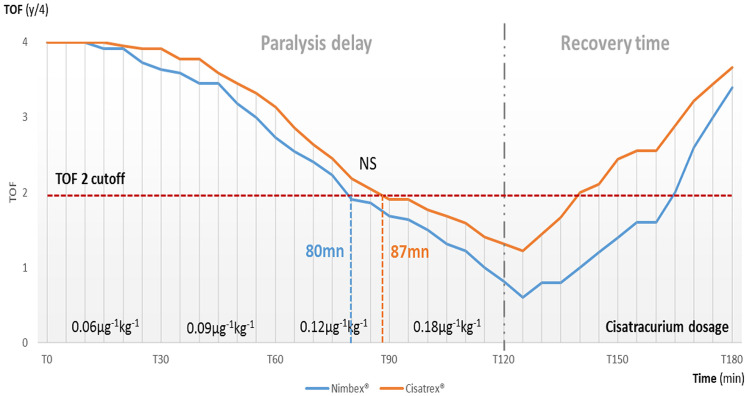
trends of the TOF in the studied patients respectively under the two forms of cisatracurium (Nimbex® and Cisatrex®) (n=22 patients); TOF, train-of-four; NS, non-significant

**Paralysis delay, recovery time and variability:** Figure 2 displays the respective curves of the evolution of the TOF with Nimbex® and Cisatrex® both for paralysis and recovery after stopping infusion. Both curves seemed to be superimposed but there is a slight but not significant late response of the Cisatrex® as compared to Nimbex® within the paralysis period while there is late response with Nimbex® compared to Cisatrex® within the recovery period. There were no statistical differences at any time of the evolution of the TOF. The time to reach a TOF at 2/4 was respectively 80min for Nimbex® and 87min for Cisatrex® which was not significantly different. The dosing regimen of 0.12mg.kg-1.h-1 was necessary to achieve a TOF at 2/4 while the dosing regimen of 0.18mg.kg-1.h-1 achieved a minimal TOF of 0.8 for Nimbex® and 1.3 for Cisatrex® within an additional time interval of 30 min. Within the 60 min of the recovery period, the between-two-studied drugs´ difference in TOF was estimated at 0.25±0.96, p=0.64. Regarding variability, at each time interval of the study periods, there were no significant differences between the two studied forms of cisatracurium either within the paralysis period or the recovery time.

**Tolerance:** the trends of heart rate (HR) and blood pressure (BP) regimens were rather stable within the 2 periods of paralysis and recovery respectively with Cisatrex® and Nimbex®. There were no significant differences between the two drugs. The slight variations didn´t reach the pre-defined cutoff of 30% increase from the baseline values for HR (115b/min) and 30% decrease from the respective baseline value for BP (Systolic BP, 130mmHg; Diastolic BP, 75mmHg). Within the study period there was no drug interaction event especially with antibiotics.

## Discussion

The present randomized double-blind crossover study which investigated the efficacy and the tolerance of two forms of cisatracurium (brand name Nimbex® and generic Cisatrex®) demonstrated no significant differences in the paralysis, the recovery times or in tolerance in hypoxemic mechanically ventilated critically ill patients. To the best of the authors´ knowledge (relying on recent PubMed searches accessed on March 2019, between 1966 and March 2019, using the following MeSH words: cisatracurium, generic, brand, bioequivalence), the present study would be the first to address the issue related to the comparison between the bioequivalence and the pharmacodynamics properties regarding the brand-name and the generic of cisatracurium. This was a randomized double-blind crossover study. This design is highly adapted to this kind of physiological studies aimed at demonstrating differences in paralysis and recovery delays between the two studied forms of cisatracurium [26, 27].

In the present study, the calculated sample size estimated at 22 is satisfactory compared to several studies including samples ranging from 6 to 37 patients [15, 23, 24, 28-32] (Table 3). All patients presenting an indication for paralysis within the study period were included. Indeed, the sample was featured by a large proportion of COPD patients compared to the more classical indication mainly ARDS patients [6]. The relatively high proportion of COPD patients is explained by the particular recruitment of this spectrum of severe AE/COPD patients presenting at ICU admission with a clinical picture characterized by a severe obstructive disorder generating major patient ventilator asynchrony. The relatively low median PEEP (positive end-expiratory pressure) may be explained by the important proportion of COPD patients in whom PEEP was set at zero. Indeed, this could be revealed by the elevated median autoPEEP.

**Table 3:** studies from literature dealing with the dosing regimens respectively used to achieve neuromuscular blockade and recovery time

Author, Year	Design	N	Objective	Dose of cisatracurium	Recovery time(min)^*^ Recovery index^**^
Prielipp *et al*. [24] 1995	Prospective randomized double blind multicenter	28	Recovery time cisatracurium vs. vecuronium	Mean infusion 0.15mg.kg–1.h–1	68±13^*^
Boyd *et al*. [15] 1996	Prospective randomized clinical trial	6	PK-PD cistracurium vs. atracurium	Bolus 0.1mg.kg–1.h–1 mean infusion 0.19mg.kg–1.h–1	60 (20-85)^*^
Pearson *et al*. [23] 1996	Prospective randomized, single-blind study	12	Infusion requirement nd recovery of ciasatracurium vs. atracurium	Bolus 0.1mg.kg–1.h–1 Mean infusion 0.23mg.kg–1.h–1	46 (8.3-64)^*^
Lagneau *et al*. [18] 2002	Open labeled multicenter prospective randomized study	102	TOF 2/4 vs. 0/4 of continuous infusion of cisatracurium	Mean infusion 0.21mg.kg–1.h–1	-
Baumann *et al*. [22] 2004	Prospective randomized	30	TOF vs. clinical assessment	Mean infusion 2.3±0.2µg.kg–1.h–1	45±7^*^
Burmester *et al*. [28] 2005	Prospective randomized double-blind	37	Vecuronium vs. cisatracurium contiunuous infusion in children ICU	-	-
Forel *et al*. [29] 2006	Prospective multicenter randomized trial	36	NMBA on gas exchange	Bolus, 0.2mg.kg–1 continuous infusion at an initial rate of 5µg.kg–1.h–1	-
Papazian *et al*. [30] 2010	Multicenter double-blind trial	340	Clinical outcomes 2 days of NMBA in ARDS patients	-	-
Dong *et al*. [31] 2012	Prospective experimental	30	PD of cisatracurium continuous infusion vs. bolus injection	-	13.13±3.36^**^
Dieye *et al*. [12] 2014	Prospective observational study	34	Cisatracurium required doses in MICU vs. SICU	0.15mg/kg followed by repeated boluses of 0.03mg/kg every four minutes.	-
Diaz *et al*. [32] 2014	Prospective experimental	10	Potency, onset and recovery cisatracurium vs. CW002 and pancuronium	multiple intravenous doses starting at 0.015mg.kg–1	5.6±0.8^**^

The TOF, used in the present study to monitor the paralysis in response to both studied drugs, is one among the most commonly used methods [33]. Indeed, the TOF proved to be one of the most balanced tools between the monitoring of the paralysis delay and the recovery time [20]. Typical fade of the TOF response defines competitive NMB by nondepolarizing NMBAs. The cutoff of TOF below 2/4 used to define the paralysis delay was chosen according to the study by Lagneau *et al*. [18], which demonstrated the clinical relevance and safety of this cutoff when compared to a TOF at 0/4. These data were thereafter confirmed by the 2002 “Clinical Practice Guidelines for Sustained Neuromuscular Blockade in the Adult Critically Ill Patient” updated in 2016 [19, 34].

This study presents three limitations. The first limitation concerns the difficulty to assess sharply the TOF recovery time, in a narrow spectrum of patients presenting rather decreasing in the amplitude of contractions. The co-investigator (NF) was well trained to this task especially via the pre-protocol patient. Otherwise, the crossover design was sufficient to offset this limitation. The second limitation is, when introducing cisatracurium, a bolus is usually administered before the continuous infusion [12, 15, 23]. This bolus has not been used in the protocol to allow a progressive decrease of the TOF and thus the comparison of the decline of the TOF and the paralysis time delays to reach a TOF of 2/4. One could rather consider the design Bolus 1- Monitoring of Recovery - Washout period - Bolus 2 - Monitoring of Recovery to be interesting at least in regard of the aim of the study. Two arguments could be developed for this. First, as clinical considerations, patients with oxygenation deficit (severe ARDS) as well as decarboxylation deficit (AECOPD, Asthma) and need of relaxation to improve ventilation will definitely gain profit from an immediate relaxation, rather than a progressive one displayed over two hours with low starting doses, increased thereafter. Second, as pharmacological considerations, the wash out of middle-long NDMR (non-depolarizing MR) like cisatracurium are more Dose-Dependent and less Time-of-Application-Dependent (when given continuously). That means by the time of stopping the IV-application, starts the wash out period, which is about (45-50 min) [15, 22-24].

The problem is, a clinical TOF-recovery is not linear to the pharmacological recovery. Most current NDMR from Benzylisochinolin family or from Steroidal family have, a) a dose-effect-ratio around 70-75%, which means that 70-75% of the Acetylcholine receptors (ACh-receptors) have to be engaged/bound to have a clinically apparent muscle relaxation. b) an autonomic-safety-reserve (= the ratio of neuromuscular blocking-dose needed to expect the muscarinic effect = dose needed to effect observed) set up high (Example 20: 1 for vecuronium) in comparison to depolarizing MR or old MR. Meaning that 20 time the MR dose needed for the effect is given IV. This is to have a reserve in deploying the MR effect [35]. Both effect 1 and 2 means in reverse conclusion, that an observed clinical recovery in muscle relaxation (TOF-based) after one hour can just mean that 30% of the receptors are free, but maybe most of them are still bound by the MR. This has normally no clinical relevance in intensive care (compare to anesthesiology or emergency care), but applying a new drug after this one hour, based on the sole clinical recovery can lead to interaction between the rest-MR of the first arm of the study, and the second one. The blood levels of the first medication has not been sampled prior to the application of the second MR in this study, so that it remains unclear at what pharmacological levels of the first the second one has been applied, and interact with the first one. Actually, in clinical routine, this must NOT be done, but in respect to the kinetic of MR-binding biology, a greater wash-out period would have reduced the risk of drug interactions, which can bias the results in a cross-over design. The random order could, although, have lessened the difference.

Another possibility could have been to start with a bolus followed by a predefined low continuous infusion regimen (i.e. 0.06mg.kg^-1^.h^-1^) for the two studied drugs, then monitor the TOF increase. But we have to define the dosing regimen, and the effect of the bolus could largely delay the procedure and thus alter the benefit of the cross-over design. The third limitation regarding the monitoring of the paralysis, is that clinical parameters, ventilatory parameters and patient-ventilator asynchronies have been just studied to ascertain that patients presented no significant asynchronies and were clinically well paralyzed. This was naturally because of the physiological design of the study.

**Paralysis delay:** in this study, there was no differences in the paralysis delay between the two studied forms of cisatracurium. Within this period, the increments of continuous infusion dosing regimens were similar ranging from 0.06 to 0.18mg.kg-1.h-1. Indeed, the paralysis delay of respectively 80 and 87 min was quite comparable to literature data. Boyd *et al*.[15], in a clinical trial including 12 critically-ill patients, comparing pharmacodynamics and pharmacokinetics of an infusion of cisatracurium or atracurium, demonstrated a paralysis delay to reach a TOF of 0.7 of 70 min. This delay was achieved with an infusion rate of 0.19mg.kg-1.h-1.

**Monitoring:** the target of 2/4 of TOF was chosen for its clinical relevance based on the guidelines [19, 34]. Three levels of NMB were defined and suggested that patients presenting with ventilator asynchrony may be stabilized with a partial paralysis achieving a TOF of 2/4. Lagneau *et al*. [18] in an open labeled multicenter prospective randomized study including 102 mechanical ventilated patients with PaO2/FiO2 <200mmHg, compared two levels (2/4 vs 0/4) of continuous cisatracurium induced curarization and concluded that a blockade at 2/4 of TOF has a similar effect on respiratory parameters, plateau pressure (Pplat) and PaO2/FiO2 as a blockade at 0/4 allowing a decrease in total administrated doses and a shortening of the muscle-strength recovery after stopping the infusion. In a recent study, Bouju *et al*. [6] revealed a huge discrepancy between the clinical assessment and TOF and stated that a TOF of 1 to 2/4 is a goal rarely achieved at usual doses of NMBAs. They even added that respiratory objectives for Pplat and oxygenation could be achieved in ARDS patients without TOF monitoring in a prospective descriptive study including 119 patients. Baumann *et al*. [22], in a prospective randomized clinical trial including 30 patients with ventilator asynchrony, demonstrated no differences in outcomes (post paralytic mean recovery times) between clinical assessment and TOF monitoring. Groetzinger *et al*. [36], in a retrospective review in 378 ARDS patients, failed to demonstrate any correlation between TOF, ABG´s parameters or even oxygenation index.

Recently Hraiech *et al*. demonstrated in a prospective open labeled study conducted in ARDS patients that they were able to decrease cisatracurium consumption with a nurse-driven protocol based on TOF monitoring for NMBA administration without significantly affecting the quality of the neuromuscular block [37]. There are increasing recommendations to use quantitative TOF in monitoring neuromuscular blockade [38]; because, a qualitative TOF of 2/4 can mean many different underlining bloods-levels of a middle-long MR, and a clinical recovery just means that at least 30% of receptors are free. This is not an issue in regards to the setting of the present study within intensive care patients, who do not need a fully recovery at this level of intensive care management. But, in regards to the conclusion and recommendation to use Cisatrex® as good as Nimbex®, in line with a TOF-recovery, this should be put into perspective in the setting of Anesthesiology and Emergency Care.

**Variability:** it was noteworthy to observe that in both studied drugs there was no variability in the paralysis effect all across the paralysis period in response to the consecutive three increments of dosing regimens after the initial dose of 0.06mg.kg^-1^.h^-1^. This feature was already demonstrated in the study by Xiaobo *et al*. [9]. Comparing rocuronium versus cisatracurium in a prospective randomized trial including 80 patients under total intravenous anesthesia. Authors, demonstrated that cisatracurium showed less variability either in the duration of the mean recovery time when compared to rocuronium.

**Doses:** in the present study, regular increments were chosen every 30 min to reach the final dose of 0.18mg.kg-1.h-1 starting at the initial dose of 0.06mg.kg-1.h-1. This regimen was namely based on the 2002 “Clinical Practice Guidelines for Sustained Neuromuscular Blockade in the Adult Critically Ill Patient” updated in 2016 [19, 34] using patient weight at admission and suggesting the dose of 0.15mg.kg^-1^.h^-1^. Table 3 displays several studies with dosing regimens ranging from 0.15 to 0.50mg.kg^-1^.h^-1^.

**Recovery time:** the present study demonstrated no significant difference in the recovery time between the two forms of cisatracurium. This delay was estimated at 60 min as in the study by Newman *et al*. [39] which demonstrated similar recovery time to TOF 70% (of the initial TOF value) in both cisatracurium 62 min vs atracurium 60 min. Similar results did not find any differences for the recovery time to TOF 70% (45 vs 46 min) [23]. In another study Prielipp *et al*. [24], comparing cisatracurium to vecuronium, found recovery times of TOF 70% respectively at 68 and 87 min. In the same study by Lagneau *et al*. [18], recovery time from NMB was significantly longer in the TOF group of 0/4 than in the TOF group of 2/4 (75 vs 45min, p=0,0008) (Table 3).

**Side effects:** within the short period of the study, required by the crossover design, only the hemodynamic tolerance of the two forms of cisatracurium has been assessed. Indeed, it would have been impossible to search for interactions with antibiotics for instance. Furthermore, the present study identified no significant cardiovascular disorders. Diaz *et al*. [32], in a crossover experimental study including 10 New Zealand white rabbits, identified no significant hemodynamic instability with cisatracurium compared to pancuronium. Papazian *et al*. [30] described one patient who developed bradycardia during cisatracurium infusion. Lagneau *et al*. [18] reported one or more adverse events in 32 out of 132 patients. Authors identified 12 events as considered to be drug related in 10 patients and only one presented arrhythmia.

## Conclusion

The present study demonstrated the equipotent effect of two marketed forms of cisatracurium (brand name Nimbex® and generic Cisatrex®) in hypoxemic mechanically ventilated critically ill patients. Indeed, both forms displayed equivalent paralysis delays and recovery kinetics. Tolerance was also equivalent as demonstrated by the absence of significant variations of cardio-circulatory parameters.

### What is known about this topic

- In developing countries as in many others, cisatracurium-use remained rather modest because of economic considerations;
- Recently the introduction of Cisatrex®, the generic of the brand name Nimbex®, provided an opportunity to substitute cisatracurium to other neuromuscular blocking agents (NMBAs);
- Physicians’ reluctance to prescribe generics may impede their use, because of all the surrounding controversies, involving generic drugs legislation especially the approval process, issues of bioequivalence and corruption that have arisen.

### What this study adds

- Authors hypothesized that a proof of safety and efficacy of Cisatrex® compared to its original brand-name could be of an invaluable support to reassure the intensivist while using Cisatrex®
- This is a crossover double-blind randomized study to compare efficacy and tolerance of two marketed forms, brand-name (Nimbex®) and generic (Cisatrex®) of continuous cisatracurium-induced paralysis in hypoxemic ventilated patients in a medical intensive care unit;
- The present study revealed no significant differences in efficacy as in tolerance between brand-name Nimbex® and generic Cisatrex® of cisatracurium continuous infusion in hypoxemic ventilated patients.
